# Clinical decision making in the recognition of dying: a qualitative interview study

**DOI:** 10.1186/s12904-016-0179-3

**Published:** 2017-01-25

**Authors:** Paul Taylor, Dawn Dowding, Miriam Johnson

**Affiliations:** 1 St Benedict’s Hospice, St Benedict’s Way, Sunderland, SR2 0NY UK; 2 Columbia University School of Nursing and the Visiting Nurse Service of New York, 617 W 168th Street, New York, NY 10032 USA; 3 Hull York Medical School, University of Hull, Hertford Building, Hull, HU6 7RX UK

**Keywords:** Dying, Diagnosis, Clinical decision making

## Abstract

**Background:**

Recognising dying is an essential clinical skill for general and palliative care professionals alike. Despite the high importance, both identification and good clinical care of the dying patient remains extremely difficult and often controversial in clinical practice. This study aimed to answer the question: “What factors influence medical and nursing staff when recognising dying in end-stage cancer and heart failure patients?”

**Methods:**

This study used a descriptive approach to decision-making theory. Participants were purposively sampled for profession (doctor or nurse), specialty (cardiology or oncology) and grade (senior vs junior). Recruitment continued until data saturation was reached. Semi-structured interviews were conducted with NHS medical and nursing staff in an NHS Trust which contained cancer and cardiology tertiary referral centres. An interview schedule was designed, based on decision-making literature. Interviews were audio-recorded and transcribed and analysed using thematic framework. Data were managed with Atlas.ti.

**Results:**

Saturation was achieved with 19 participants (7 seniors; 8 intermediate level staff; 4 juniors). There were 11 oncologists (6 doctors, 5 nurses) and 8 cardiologists (3 doctors, 5 nurses). Six themes were generated: information used; decision processes; modifying factors; implementation; reflecting on decisions and related decisions. The decision process described was time-dependent, ongoing and iterative, and relies heavily on intuition.

**Conclusions:**

This study supports the need to recognise the strengths and weaknesses of expertise and intuition as part of the decision process, and of placing the recognition of dying in a time-dependent context. Clinicians should also be prepared to accept and convey the uncertainty surrounding these decisions, both in practice and in communication with patients and carers.

## Background

There is an increasing emphasis on end of life care, including how and where patients die [1, 2]. There have been considerable concerns raised regarding the quality of care provided to patients at the end of their life in hospitals, and also issues related to their place of death [3]. This raises particular issues for clinicians; they need to be able to recognise whether a person is dying (or not) in order to ensure effective symptom control, to inform decisions regarding an appropriate ceiling of medical intervention, consideration of potentially reversible issues, and communication with patients and their loved ones [4]. One of the key recommendations or priorities for care across a number of initiatives that have been introduced nationally in the UK to improve end of life care [5, 6], is that clinicians “recognise and communicate that a person is dying.” However the unpredictable nature of the trajectory of a disease means that it is often difficult for clinicians to anticipate when a patient may die [7].

In the UK, up until 2013, care of the dying was codified and structured in the Liverpool Care Pathway (LCP), and this document included simplified advice on recognising dying. The final version advocated a multi-disciplinary decision process, regular reviews of patient condition and highlighted clinical signs which were useful but not definitive [8]. Following controversy over the implementation of the LCP [9], the independent Neuberger review was commissioned which ultimately led to its withdrawal [10]. One concern highlighted in the review was the lack of evidence surrounding the recognition of dying.

Three and a half years since the publication of the Neuberger Review, the LCP has not been replaced by a single national system of comparable scope. Locally, trusts and health care organisations have attempted to generate structured processes for end of life care, whilst seeking to avoid the reported flaws of the LCP. Nationally, a number of related initiatives exist which aim to a greater or lesser extent to support end of life care, some of which make reference to the withdrawal of the LCP [5, 6]. In each of these initiatives, the recognition of dying is highlighted as important; this landscape is summarised in Table 1.

**Table 1:** Pertinent examples of ongoing documents and initiatives relating to care of the dying

Document/initiative	Brief description	Reference to recognising dying
NHS End of Life Care Strategy [33]	National framework and vision for end of life care in the NHS; remains a foundation document in context of changing landscape.	Identifying people approaching the end of life is highlighted as one of seven key areas relevant to commissioning and delivering care.
The Liverpool Care Pathway version 12 [8] (now withdrawn).	Previous national guidance on end of life care. Now withdrawn.	Recommended a multi-disciplinary approach with regular reviews of decisions and patient condition.
The route to success – transforming end of life care in acute hospitals [38].	Original document supporting NHS managers and clinicians responsible for delivering end of life care. Latest version in progress. “How to” guide below.	Reinforces points made in the End of Life Care strategy, above; “Early recognition that a patient is dying is a key element in quality end of life care.”
Transforming end of life care in acute hospitals – The route to success “how to” guide [39]	Latest advice on implementing “The route to success” document, above. Draws multiple resources together to produce a coherent guide.	Repeated references as cited in other resources.
More Care, Less Pathway [10].	The independent review into the LCP.	Highlighted that failure to recognise dying accurately was a key weakness of implementing the LCP
One Chance to Get it Right, Five priorities for end of life care [5].	Document summarising recommendations from the Leadership Alliance for the Care of Dying People (LACDP). Summarised in Five Priorities for end of life care.	First of five priorities cited is to “recognise and communicate that a person is dying”. Complexities and challenges recorded as part of discussions.
National Institute for Health and Care Excellence (NICE) Guidance: Care of dying adults in the last days of life [6].	National guidelines on end of life care.	First section is advice on recognising dying, with discussion of challenges and complexity of decisions.
		First research recommendation relates to recognising dying.
Ambitions for palliative and end of life care: A national framework for local action: 2015–2020 [40].	Document produced by a partnership of organisations, outlining ideals and targets for improving palliative and end of life care in England.	Acknowledges the difficulty and uncertainty in recognising dying, and the importance of honest discussions as part of individualised care (Ambition 1)
National Care of the Dying Audit for Hospitals, England: National Report May 2014 [1].	Document summarising audit of end of life care in England in 2014. Made key recommendations, based on audit findings and results of Neuberger review.	Recommends that recognition of dying is undertaken by the multidisciplinary team and communicated to patients/families.
Actions for end of life care: 2014–2016 [41].	Intended as a document revisiting and refreshing the end of life care strategy (above).	Includes a commitment to work with organisations to improve ability of professionals to recognise dying. (Section 5.2, commitment 10).
AMBER care bundle [42]. (AMBER is an acronym: Assessment, Management, Best Practice, Engagement, Recovery Uncertain)	A decision-making tool supporting advance care planning and setting ceilings of care in unwell patients. Recommended as part of Ambitions for palliative and end of life care (above)	Part of the role of the tool is to explore appropriate actions in event of deterioration, to guide end of life vs acute care; recognition of dying is therefore implied.

Within one month of the final withdrawal date for the LCP, some of these local and national initiatives had already met with criticism from the same sources as the original concerns [11]. Whilst these were presented in the mainstream press, recognition of dying featured again.

Given the importance of being able to accurately recognise whether or not an individual is dying, to inform management decisions and enable patients to make informed choices, the evidence base for the recognition of dying is limited [12]. Historically, a number of patient signs have been used as indicators of impeding death, including profound weakness, a patient being bed-bound or comatose, only able to take sips of fluid, changes in breathing pattern/breathlessness, skin changes, weak pulse and falling blood pressure [13]. However, the evidence base for these signs is limited; there is no research that provides an overview of the strength of association between the signs and time of death. Furthermore, they may not all be exclusively associated with dying; they may also be signs of an acute and potentially reversible illness [14]. Whilst these signs may be useful when recognising death, therefore, they are not helpful in assisting with distinguishing the dying patient from a patient who has a potentially treatable illness.

In summary, whether developing public policy, undertaking research, performing clinical care, or representing the views of the public, recognition of dying is repeatedly highlighted as a keystone to good end of life care. Despite, however, the high importance of the skill, it remains extremely difficult and there are large margins of error in the ability to recognise the final days of life in terminally ill patients, both from experts and well-designed prognostic models [15, 16]. As Table 1 highlights, there are ongoing calls for research into this domain, and multiple sources highlighting the challenges involved. As yet, however, no clear headway has been made.

One alternative view of the recognition of the dying patient, is to treat the decision process as one of prognostication; the action of making predictions about future events (such as survival). Overall, clinicians have been shown to be inaccurate in their predictions of survival; studies compare a clinician’s prediction of survival (CPS) with actual survival (AS) identifying errors such as the patient living longer or shorter than predicted. A review of such studies carried out by Glare in 2003 [17] indicated that clinicians overestimated survival in 27% of cases and underestimated in 12%. Prognostic models (developed from cross-sectional studies of signs and survival) may also provide valuable insights and potential associations between patient signs and their likelihood of dying. In a recent study, the authors identified a number of specific physiological changes over the last two weeks of life for patients with cancer; including deterioration in respiratory function, worsening renal function (as measured by abnormal blood markers) and changes in serum albumin (with more abnormal values as death approaches) [18]. Whilst the identification of such physiological markers may help with the accurate recognition of a patient close to death; what is unclear is if, or how, clinicians may be able to incorporate them into their decision making.

### Decision-making in health care

Decision-making is a branch of psychology, overlapping with cognitive science and sociology, which is concerned with understanding, modelling and improving decision-making processes [19], made under conditions of uncertainty [20]. Decisions made by health care professionals are normally uncertain; the information that is used to inform decisions is often incomplete, and the outcomes are based on probability [21]. There are three main approaches to exploring decision making; normative, prescriptive or descriptive [22]. Normative approaches are concerned with mathematically modelling decisions and outcomes [23], prescriptive approaches focus on generating techniques and tools to improve decision-making in the real world, and often draw on normative models [24]. Descriptive approaches are concerned with decisions as made by human decision-makers, particularly where they deviate from normative predictions [25]. Studies have demonstrated, for example, the role of decision-making models in prognostication [26] and decisions around treatment withdrawal [27].

There are a number of descriptive theories of decision making [24], in this paper we focus on the approach most relevant to understanding potential prognostic decision processes for recognising when a patient is dying. Dual process theory suggests that individuals make decisions (reason) using two different types of cognitive process; System 1 thinking, which is fast and intuitive and system 2 thinking, which is slow, analytical and thorough [28]. System 1 thinking is the default approach to thinking, it can process large amounts of data rapidly and does not require the use of much working memory. Akin to ‘intuition,’ it is a reasoning process that is often used by experts, and is triggered by ‘context’; with expert clinicians potentially identifying specific patterns or cues and matching them to previous examples of the same patient, based on their extensive experience. In contrast, system 2 thinking is characterized by being conscious, controlled and rule based. In dual process theory it is thought to provide a ‘supervisory’ role, regulating system 1 thinking, and promoting more systematic approaches to decision making [29].

When making decisions in healthcare settings, a number of properties of the decision-maker have the potential to influence the decision process, and relate to the system 1/system 2 distinction. Seniority maps to experience and expertise in a given context. Profession (doctors compared with nurses) also influences the process [30]. Patient diagnosis may also influence the recognition of dying, as cancer and organ failure may be considered to follow distinct trajectories as death approaches [7].

This paper reports the results of a qualitative study that explored the clinical decision processes of healthcare professionals who are faced with the need to recognise if and when a patient may be dying.

### Aim

The aim of the study was to explore current decision processes in the recognition of dying, to inform potential strategies for implementing the results of prognostic models into practice to support that decision process.

## Methods

This was a qualitative study, using semi-structured interviews, to explore the decision processes around recognising dying with clinical staff working in oncology and cardiology units in England.

The study was conducted with medical and nursing staff in a hospital in England which contained a referral centre for both cancer and cardiology. This hospital was a National Health Service (NHS) trust; a service overseen and funded entirely via the Department of Health. Staff who participated in the study were drawn from the oncology unit and cardiology unit. The oncology unit consisted of five dedicated wards, with 18 haematology/oncology patients on each. The unit included dedicated high dependency and palliative oncology beds. The cardiology unit included 2 wards with a total of 47 general beds and 19 coronary care beds. Each unit accepted patients at all stages of their illness, including acute admissions directly from the community, admissions for specialist intervention, referrals from general admissions/Emergency Department to treatment support and for palliation.

### Participants

Purposive sampling was used to identify potential participants. A sampling frame was derived (see Table 2) to address three personal factors demonstrated in the palliative medicine and/or decision-making literature to have potential to influence clinical decisions surrounding the end of life: profession (doctor or nurse), specialty (cardiology or oncology) and grade (senior vs junior).

**Table 2:** Purposive sampling frame. Numbers in brackets indicate number recruited in each category

	Oncology		Cardiology	
	Doctors	Nurses	Doctors	Nurses
Senior	Consultant (2)	Ward sister/Matron (2)	Consultant (1)	Ward sister/Matron (2)
Intermediate	ST3+ (2)	Staff nurse (2)	ST3+ (2)	Staff nurse (2)
“Junior”	FY1/2 (2)	HCA (1)	FY1/2 (0)	HCA (1)

Notes on UK grades and abbreviations: Consultant: Most senior grade of doctor; equivalent to attending physician. ST: Specialty trainee; a doctor training to become a consultant in a specialty. FY: Foundation Year; a doctor typically 1–2 years post qualification. Sister/Matron: Most senior grades of nurse. Staff nurse: Qualified nurse with degree-level training. HCA: Health Care Assistant; nurse trained through experience; also termed auxiliary nurse and nursing assistant

The initial aim was to recruit one to two participants per factor until saturation of themes (assessed by iterative ongoing analysis) was obtained. Potential participants were recruited to the study through the use of posters, letters and through presentations at teaching sessions to invite staff to take part in the study. Interested participants approached the lead researcher, through direct discussion or telephone, to ask to take part.

### Data collection

An interview schedule was designed, based on decision-making literature, and used to prompt and guide data collection. During the interview the participant was asked to bring to mind a specific decision, and then to base their responses around that case, in order that the decision processes could be explored in detail. The interview began with broad, open questions, designed to allow the participant to volunteer information as they felt appropriate, and then moved to focus on the context for the decision, information sought as part of the decision process, the decision process itself, the management of the patient and finally the potential for this decision to impact on future work. Through this approach the interview was designed to cover the full decision process.

Each participant attended for a single interview, which was digitally recorded and verbatim transcribed. Contemporaneous hand-written field notes were kept and added to the transcripts at a later date.

### Data analysis

Data were analysed using thematic analysis as proposed by Braun and Clarke [31]. This includes analysis in six stages; familiarization with data, generate initial codes, search for themes, review themes, define and name themes, produce report. These stages as applied to this research are described in further detail in Table 3.

**Table 3:** Short summary of Braun and Clarke’s approach to thematic analysis, as applied to this study

Stage	Description	Notes for this study.
1) Familiarisation with data	The researcher is immersed in the data, through repeated exposure.	Took place through interview conduct, transcribing interviews, repeatedly reading the transcripts whilst listening to the recordings, and later annotating transcripts whilst reading.
2) Generating initial codes	The researcher begins to document a list of codes, beginning during familiarization. Codes identify a piece of data that conveys meaning.	A code list was kept from early on in the familiarization process, and codes were accorded a clear definition.
	Codes are attached to the data at the point at which they arise.	Atlas.ti was used to link codes and transcript data.
3) Searching for themes	The researcher seeks common themes that unite codes. Themes are units of analysis and interpretation.	Any potentially interesting themes were considered, which united multiple codes.
4) Reviewing themes	An iterative process by which themes are explored and reviewed in detail, to determine the extent to which they may be supported by the data. Themes may be kept, combined or rejected at this point.	This was the most involved stage of the research. Analysis of double-coded transcripts, described in the text, formed an important part of this process.
5) Defining and naming themes	Following the above stage, themes are defined and named in a manner that accords meaning clearly and succinctly.	In this study, the defined themes are used for the discussion presented below.
6) Produce report	A detailed reflexive discussion of the overall process, based around the final thematic list, is generated.	Summarised in this publication.

All data were managed using Atlas.ti software (version and reference). All transcripts were coded by the lead researcher (PT), with four transcripts (one doctor and one nurse from each specialty) double-coded by a second researcher (DD) using the coding frame generated in stages 1 and 2. This allowed triangulation of findings and aided with decisions to maintain and reject codes and themes which were well or insufficiently rooted in data – areas of agreement and disagreement between coders were key to this process. Two participants (one from each profession) were contacted following analysis and met to discuss the findings and themes, providing respondent validation.

## Results

**Fig. 1:**
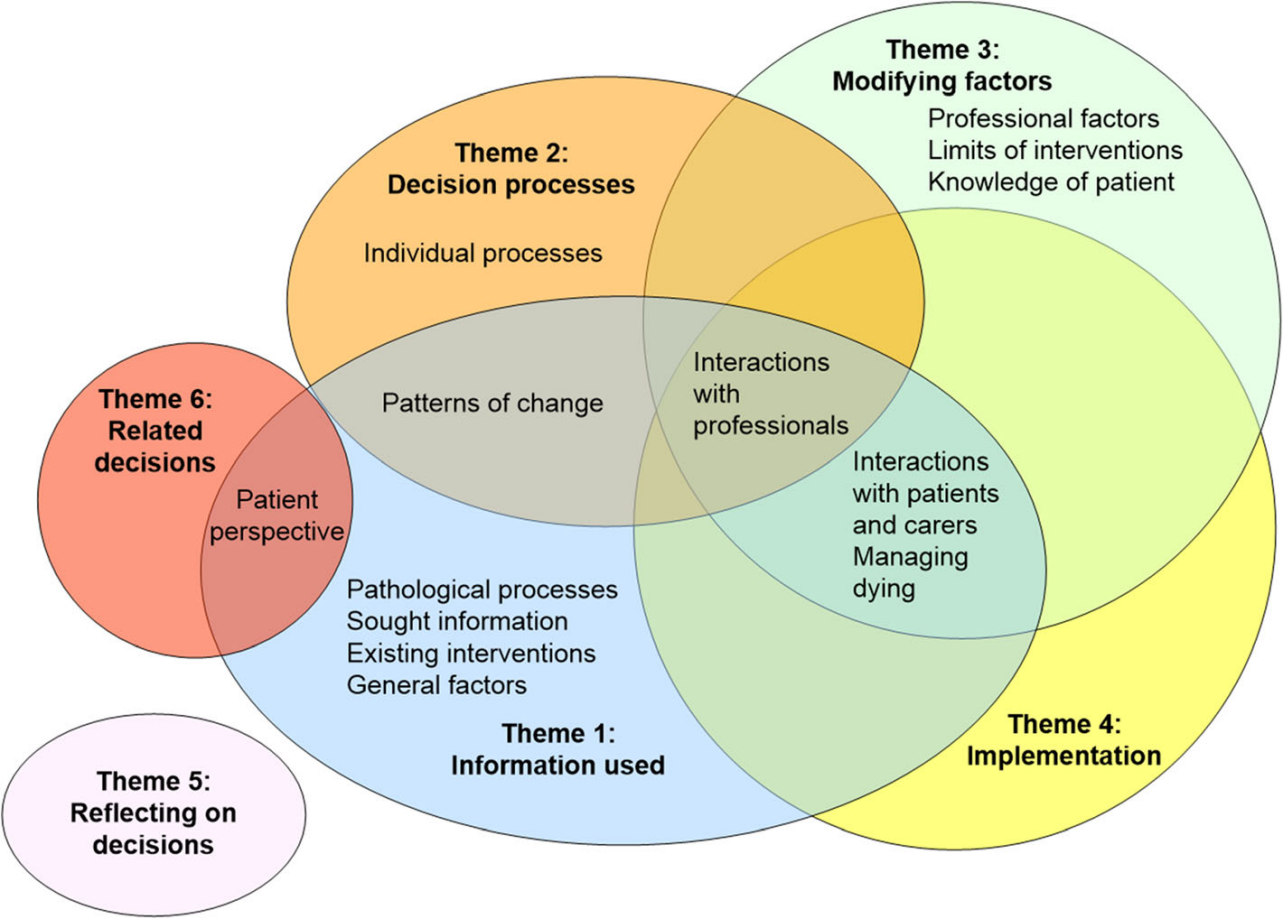
Representation of the six main themes, thirteen sub-themes and their overlap.

A total of 19 respondents; 9 doctors, 8 registered nurses and 2 health care assistants took part in the study (Table 2). Interviews varied in length from 24 to 55 min. Overall, six themes representing factors that influence the recognition of dying were generated from the analysis; information used, decision processes, modifying factors, implementation, reflecting on decisions and related decisions. Within these six themes, thirteen sub-themes were identified, some of which overlapped between themes. These are presented in Fig. 1 and described further below.

Results are presented according to themes, with supporting quotes where appropriate. Respondents are identified according to the characteristic of interest for the quote.

### Information used

Respondents collated a wide variety of information as part of their decision process when they were identifying if a patient was dying. This included knowledge of the underlying illness (both in a given patient’s case and in general), symptoms, observations, response to treatments, and non-specific factors such as mood, performance status and general fitness. The variation in responses across interviewees was considerable; specific symptoms, observations and tests reported by at least one participant are listed in Table 4.

**Table 4:** Information used as part of recognising dying

	Symptoms and signs		Other information	
Cancer	Breathlessness (7)	Pain (2)	“Observations” (4)	Imaging (4)
	Difficulty with oral intake (6)	Incontinence (1)	Oxygen saturations (3)	Biochemistry tests (3)
	Reduced conscious level (6)	Agitation (1)	Hypotension (3)	“Bloods” (2)
	Bedbound (4)	Clamminess (1)	Respiratory rate (3)	Haemoglobin (1)
	Skin colour (4)	Cachexia/loss of muscle bulk (1)	Urine output (2)	Albumin (1)
	Respiratory tract secretions (3)	Weight loss (1)	High temperature (1)	White cell count (1)
	Other respiratory changes (3)	Increased dependence (1)	Bradycardia (1)	CRP (1)
	Increased sleep (3)	Anxiety (1)	EWS (1)	Blood cultures (1)
	Confusion (3)	Jaundice (1)	Chest drain output (1)	ECG (1)
	Fatigue/energy level (3)	Ascites (1)	Specific instance not to use observations (1)	
	Nausea/vomiting (2)	SVCO symptoms (1)		
	Reduced responsiveness (2)	Odour (1)		
	Weakness (2)	Headache (1)		
	Cool peripheries (2)			
Heart failure	Breathlessness (5)	Housebound (1)	Body weight (3)	Biochemistry tests (4)
	Difficulty with oral intake (4)	Oedema (1)	Hypotension (3)	“Bloods” (2)
	Increased dependence (3)	Confusion (1)	Urine output (3)	Echocardiography (2)
	Weight gain (3)	Increased sleep (1)	“Observations” (2)	ABG (1)
	Reduced conscious level (2)	Ascites (1)	Glasgow Coma Scale (1)	CT head (1)
	Other respiratory symptoms (2)	Pain (1)	Tachycardia (1)	Serum sodium (1)
	Cachexia/loss of muscle bulk (2)	Skin colour (1)	Fluid balance (1)	
	Bedbound (2)	Fatigue (1)		
	Unable to express wishes (1)			

Figures in parentheses indicate the number of times each example was grounded in the data by a unique participant

*SVCO* Superior Vena Cava Obstruction, *EWS* Early Warning Score, *GCS* Glasgow Coma Scale

*“I’ve seen a patient with a kidney cancer spread to the spine. He’d just been diagnosed, just been started on sunitinib which is the standard treatment for kidney cancer and he was admitted for upper GI bleed. I discussed with the consultant and we both felt that this patient has still got at least 11 months to 12 months to live on average…*

*…In other patients it will be a different scenario, pancreatic carcinoma with liver metastases progressing on treatment, elderly chap with lots of co-morbidities. He was admitted for maybe progressive ascites and he generally looks unwell, then you start thinking towards poor prognosis, DNAR and maybe not that long to live.”*

Oncology doctor.

In addition to seeking one-off information on a patient’s case, participants described seeking time-dependent information; this was a finding that showed a difference between cancer and heart failure patients, with the specific pattern of change varying between the two. This varied according to the disease condition; patients with heart failure were considered to have a less predictable pattern of deterioration, whereas cancer patients tended to follow a pattern of gradual deterioration over time. The manner in which a person’s condition had changed, and the rate of such change, was accorded high importance, particularly by experienced professionals.

*“In my mind, I plot a gradient, and people tend to follow that gradient…*

*…you know, if somebody’s only deteriorating slowly, they’ve probably got fairly slowly progressive disease, if somebody is dropping off their perch very fast, then they’re probably going to continue to do that, so you’ve got less time to work on things.”*

Oncology doctor.

*“The trajectory of heart failure in patients who have advanced disease who are slowly deteriorating I always think is a bit like that child’s game where you try and skip a stone across the lake, you know that? So the first bounce is quite a big one and then the second one is a bit smaller and the third one is a bit smaller and the fourth one and it goes de, de, de, and then sinks and heart failure is a bit like that towards the end.”*

Cardiology doctor.

### Decision processes

The processes by which a clinician arrived at the recognition that at a patient may be dying were characterized by participants being either unable to describe their decision processes in detail, stating that such processes were difficult to explain or discussing how they recognised a ‘pattern’ which they could match to previous patients. In general participants described the process as being subconscious or a ‘sixth sense’ rather than being an explicit rational reasoning process.

*“I do have, I don’t know, a sixth sense, it’d be silly to say that, but I do kind of know when somebody’s dying.”*

Senior oncology nurse.

*“…I’m pretty certain that if you took me and 3 other random heart failure doctors and put us in a room with 10 patients, one of whom is going to die within the next 3 weeks, we’d all pick the same person. But quite putting your finger on how it is that you know that it’s that person and how soon, is more tricky…”*

Senior cardiology doctor.

*They’ll have an admission, sort them out, go home for quite a long period of time, come back, then don’t last quite so long at home, come back, don’t last quite so long at home and so it goes and there is a very recognizable pattern that people start coming in more and more often.*

Cardiology doctor

The only exceptions occurred in cases where decisions were very clear-cut or where, in one case, a participant discussed using the LCP “four criteria” [8] to recognise dying – a technique explicitly described as inappropriate by two other participants.

*“when she came in first admission that I saw her on, um, she had decreased levels of consciousness, so I guess she’d have met [the pathway criteria], she wasn’t swallowing at first, particularly well, because she was sleepy, so in theory, she might have met it, but you know, a bit of steroids and she came around, so I think you’ve got to be careful. Not just, not just use, you know, “oh well, they tick these boxes.”*

Oncology doctor.

As well as discussing the use of intuition or ‘sixth sense’ and pattern matching, all staff interviewed discussed decision making in teams. This included assessing/discussing cases with individual colleagues, sharing information in larger teams. However, there was also a sense that the eventual decision rested with senior members of the medical team.

*“Its’s more of a collaborative decision than one person, cos it’s, a lot of us around…”*

Cardiology nurse.

### Modifying factors

Modifying factors refer to the properties of the decision maker or context that impact on the decision process. There were a number of factors which were identified as influencing or modifying how individuals identified if a patient were dying, including the potential for investigations and treatments to influence outcomes, the patient’s own knowledge of their condition, and the professional’s duration of involvement with the patient. A key factor appeared to be a clinician’s medical specialty; cardiology and oncology staff recognised specific strengths and weaknesses of managing specialty-specific patients. Both oncology and cardiology staff recognised where their own specialty might be able to deal with dying patients in certain contexts.

*“I mean, the nurses on the oncology wards here, would have, if I’d been soldiering on saying “but we must do this”, and “shouldn’t we irradiate that and send him off for neurosurgery”, or whatever, they would have said, “don’t you think he might be dying?”*

Oncology doctor.

The decision-maker’s profession was also described as modifying decisions with distinctions explained as arising from time spent with patients (higher for more junior staff and for nurses) and responsibility for decisions (which ultimately were seen as resting with senior doctors). Given the importance of time-dependent change and intuition in the decision-processes, it is not surprising that time-dependent involvement and seniority are highlighted as influential.

A third modifying factor was the potential limit of interventions. This referred to clinicians looking ahead to consider whether a patient’s condition may benefit from either starting or continuing a given intervention.

*“What is important as well is to try and look for any reversible causes and I think as a clinician that it is very important to exclude that right at the very onset that whether there is anything reversible that you can do with minimal fuss and minimal intervention which would actually improve the situation for your patient to be able to receive more treatment.”*

Oncology doctor.

Knowledge of the limits of interventions, the lack of reversibility and the ability to think ahead and consider likely outcomes of interventions, were associated with ability to recognise the dying patient. Increase knowledge of the patient was also important, in terms of assessing patterns of change over time.

*I think, the longer a patient’s been with us, the easier it is to tell, because you know what the normal, what they are normally like, and whether they have deteriorated, whereas if they’ve just come in, you haven’t got as much overview of the patient really, to be able to make them choices maybe, sometimes.”*

Oncology nurse.

### Implementation

Implementation refers to actions taken once an individual recognised a patient may be dying. Recognition of dying and the management of the process were often discussed in tandem, with participants reflecting on the need for managing symptoms when someone is in the last phases of life.

“…*they generally seem to sleep a lot more. Um, they don’t respond, they certainly seem to lose interest in food, that’s what I’ve observed. And I think it’s really important that you do good mouth hygiene. I think, I think that although you don’t go in and do the observations, I think you do have to do things like, good oral hygiene and, ‘cos their mouth gets dry and horrible, and I think that’s something they don’t need to, sort of, put up with, do they, if you can keep on top of that. And, pressure area care as well…”*

Oncology nurse.

The data from this study suggests that the management of dying patients begins before dying is recognised, and recognition depended in part on response to management of deteriorating patients. In this context, the recognition of dying can therefore be seen as an iterative process. However, this overlap was not complete, and participants did discuss management alone. Key factors in the management of dying patients highlighted by participants included importance of good symptom control, good communication with patients and families, good inter-professional communication (written and spoken, including clear limits of care) and the avoidance of unnecessary interventions.

*“we took the fluids down. He had still been having some oxygen, but that was for comfort and so that he didn’t feel breathless or anything like that…. And his wife was there, and I think the other part of it was, as well as the medicines, is the supporting the wife.” Junior doctor*

### Reflecting on decisions

Reflections were explored deliberately, in order to capture the entire decision process. Participants rarely discussed reflecting on decisions, except where prompted by the interviewer. Several participants clearly stated that they had not reflected on the decision in question, and that they did not intend to do so. Other participants discussed having reflected on decisions around dying patients, but as part of a “debrief” process, and to assess whether anything could have been done differently, as outlined in the following quote.

“*I will always play back the aspects of the care that involved myself…*

*…if I did everything that I could, if I cared for the patient in the right way, if there was anything I could have done more or anything that I should have done less.”*

Oncology nurse.

One participant described reflection influencing future management; in this case, to ensure that families are always warned of the possibility of dying. In addition, several participants stated that they felt the diagnosis of dying should have been made sooner; none stated the opposite, suggesting a general tendency to make such decisions too late.

### Related decisions

Although the study was focused on the recognition of dying, participants also described other, related, decisions as part of the wider decision and management processes. The three main examples were a Do Not Attempt Cardiopulmonary Resuscitation (DNACPR) order, and recognition that a patient’s condition requires only symptomatic relief or will not recover. This theme stands distinct from the others in that, whilst it is well rooted in the data, the responses are not chiefly concerned with recognition of dying, and hence it is not explored further here.

## Discussion

The purpose of this study was to explore the process by which clinicians diagnosed dying in patients with heart failure and cancer. One of the key findings relates to the overall structure of the recognising dying decision. Rather than it being a clear, objective, one-off decision, it appears to be a fluid, ongoing and iterative process. It is ‘fluid’ because the distinction between active management and dying is blurred. It is ‘ongoing’ because the decision is not made at a single point in time but involves acquiring information over a longer time-period. It is ‘iterative’ because decision makers review their decisions as they acquire further information, whether arising out of their own observations or those of others. Interestingly, this relates to previous studies and recommendations regarding supporting and communicating end of life decisions with patients [32, 33]. The nature of this process has particular implications for the use of prognostic models to assist with the decision process of diagnosing dying; such models are normally developed on the basis of a ‘snap shot’ or cross-sectional sample of data taken at a point in time (such as 1 month or 2 weeks prior to death) and used in a static fashion to see how well individual signs are able to ‘predict’ the imminent death [34]. By their nature they are a static, one point in time approach to decision making, which is not reflected in the fluid and iterative nature of the actual decision process in practice. Given this, prognostic models may benefit from using repeated measures [35], a risk score subject to revision and a recognition of a potentially uncertain time period into their design; all features that may then mean they have more utility in clinical practice.

Our findings also suggest that the predominant reasoning method used by clinicians to recognise dying is that of intuition or pattern matching. As is typical for intuitive reasoning [36], clinicians discuss knowledge distinct from a reasoning process; even where specific factors are made known participants struggled to describe how they combine the breadth of information into a decision. However, despite the use of intuition to potentially recognise a dying patient, clinicians also described a process that seeks data over time, allowing time for a more analytical, methodical component. Our results suggest that clinicians are using a mixture of System 1 (rapid, unconscious, intuitive) and system 2 (slow, analytical) reasoning to both diagnose a patient as dying and implement appropriate management interventions. In this regard, the implementation of more structured approaches to assisting with the process (such as intelligent prognostic models and/or guidance on management interventions) would increase system 2 processing, leading to decisions that are potentially not based so heavily in intuitive or pattern matching processes.

### Study limitations

This study focused on the decisions of individual clinicians, rather than exploring the process of decision-making by teams. It is therefore unclear how the experiences of the individual clinicians may be impacted by discussion with others during team meetings and other places where decisions are taken (such as during ward rounds). Whilst it is not possible to eliminate bias from a qualitative study, several processes [37] were used to strengthen the credibility of the analysis. Firstly, the double-coding process allowed findings to be triangulated with the decision-making researcher (DD). Secondly, data that had been collected from a single focus group were used to triangulate the findings drawn from this study; no contradictory or additional information was raised from this limited dataset. Finally, the study involved respondent validation. None of the points presented here were contradicted, and no additional points were raised.

As part of reflexivity and transparency, this study should be interpreted with knowledge that the lead researcher is a palliative medicine professional and, as such, brings experience and preconceptions around the care of dying patients to the research. The lead researcher had also worked with some of the participants. In addition, he had undertaken a literature search around end-of-life decisions.

### Clinical implications

From a clinical perspective, this study supports the need to acknowledge the strengths and weaknesses of expertise and intuition as part of the decision process, and the importance of placing the recognition of dying in a time-dependent context. Clinicians should also be prepared to accept and convey the uncertainty surrounding these decisions, both in practice and in communication with patients and carers.

### Research implications

From a research perspective, this study suggests that detailed exploration of the decision process may be difficult, and that studies seeking to assess the accuracy of professional decision-making (particularly in comparison with mathematical models) should seek to do so realistically, by presenting a decision-maker with time-dependent information, and giving them opportunity to reassess and review decisions.

## Conclusions

The recognition of dying remains an important skill, highlighted by the multiple initiatives in the UK (Table 1) and the Institute of Medicine report in the USA [2]. Increased research in end-of-life care, with particular reference to the recognition of dying, are common themes. Using decision-making theories as a basis, this study explored the recognition of dying by health care professionals in cardiology and oncology, and highlighted important aspects of the decision process, which have an impact on both clinical practice and research.
